# Emerging Roles of Interleukin-33-responsive Kidney Group 2 Innate Lymphoid Cells in Acute Kidney Injury

**DOI:** 10.3390/ijms21041544

**Published:** 2020-02-24

**Authors:** Wei-Yu Chen, Lung-Chih Li, Yi-Hsiu Wu, Jenq-Lin Yang, Hong-Tai Tzeng

**Affiliations:** 1 Institute for Translational Research in Biomedicine, Kaohsiung Chang Gung Memorial Hospital, Kaohsiung 83301, Taiwan; wychen624@cgmh.org.tw (W.-Y.C.); r5239@cgmh.org.tw (L.-C.L.); martinwu19860626@gmail.com (Y.-H.W.); jyang@cgmh.org.tw (J.-L.Y.); 2 Division of Nephrology, Department of Internal Medicine, Kaohsiung Chang Gung Memorial Hospital and Chang Gung University College of Medicine, Kaohsiung 83301, Taiwan

**Keywords:** interleukin-33, acute kidney injury, group 2 innate lymphoid cells

## Abstract

Interleukin (IL)-33, a member of the IL-1 family of cytokines, is involved in innate and adaptive immune responses. IL-33 triggers pleiotropic immune functions in multiple types of immune cells, which express the IL-33 receptor, ST2. Recent studies have revealed the potential applications of IL-33 for treating acute kidney injury in preclinical animal models. However, IL-33 and IL-33-responding immune cells are reported to exhibit both detrimental and beneficial roles. The IL-33-mediated immunomodulatory functions have been investigated using loss-of-function approaches, such as *IL33*-deficient mice, IL-33 antagonists, or administration of exogenous IL-33 recombinant protein. This review will discuss the key findings on IL-33-mediated activation of kidney resident group 2 innate lymphoid cells (ILC2s) and summarize the current understanding of the differential functions of endogenous IL-33 and exogenous IL-33 and their potential implications in treating acute kidney injury.

## 1. Introduction

Interleukin (IL)-33, a nuclear cytokine belonging to the IL-1 family, has pleiotropic immunomodulatory functions and is involved in various inflammatory diseases [1]. IL-33 is expressed in various cell types, such as endothelial cells, fibroblasts, epithelial cells, and immune cells [1,2]. In humans, IL-33 functions as a tissue-derived nuclear alarmin [3,4] and is constitutively expressed in the epithelial barrier and endothelial barrier tissues [3,5]. In steady state, IL-33 is quenched within the cell through interaction with histones [6]. During apoptosis, intracellular IL-33 is inactivated by caspases like caspase-3 and caspase-7, making them immunologically silent. Unlike IL-1β and IL-18, caspase-1 mediates the inactivation of IL-33 by cleavage of its central domain [7]. Under tissue damage, IL-33 is released in its full length, which is the active form [1]. Moreover, some inflammatory proteases like tryptase from mast cells and cathepsin G and elastase from neutrophils further boost the activity of IL-33 [8,9].

The receptor complex for IL-33 comprises the ST2 subunit, which is encoded by the *IL1RL1* gene, and IL-1 receptor accessory protein (IL-1RAcP), a coreceptor [3,10]. The ST2-IL-1RAcP heterodimeric receptor is formed after binding of IL-33 ligand [11]. Both IL-1RAcP and ST2 contain toll-interleukin receptor (TIR) domain, recruiting myeloid differentiation primary response 88 (MyD88), IL-1 receptor-associated kinase-1 (IRAK-1) and TNF receptor-associated Factor 6 (TRAF6). The protein complex transmits activation signal through p38, c-Jun N-terminal kinases (JNK), and nuclear factor kappa B (NF-κB) pathways [12]. Also, a TRAF6-independent mechanism of extracellular signal-regulated kinase (ERK) activation has been demonstrated [13]. These pathways largely overlap with Toll-like receptors (TLR), IL-1 and IL-18 receptor signaling, and a ST2-specific mechanism is proposed to achieve Th2-biased gene expression [14]. This idea may be illuminated by a recent study, which suggests the activity of peptidyl-prolyl cis-trans isomerase NIMA-interacting 1 (PIN1) inducing isomerization and nuclear translocation of IRAK-M as well as triggering Th2-gene expression in dendritic cells [15]. Conversely, single Ig IL-1-related receptor (SIGIRR) is known to suppress type-2 response through TIR domain interaction with ST2 protein [16].

Recently, several studies have suggested that IL-33 is involved in the pathogenesis of kidney diseases and the associated tissue reparative responses [17,18]. For example, IL-33 level had increased in unilateral urinary obstruction (UUO) model in mice [19]. Also, a cisplatin-induced kidney injury results in increased serum levels of IL-33 [20]. Furthermore, IL-33 has been shown to be upregulated in kidney and linked to ferroptosis [21]. The elevation of IL-33 or ST2 levels in the urine has also been investigated in renal transplant [22]. Despite multiple kidney injury models resulting in the upregulation and release of IL-33, the correlation of kidney injury and clinical outcome is not unified, which may be due to the diverse pathogenic effects of IL-33 and limitations on the tools to detect IL-33 level in serum and urine samples (reviewed in Chen et al.) [18].

Acute kidney injury (AKI) is a common complication among hospitalized patients [23]. AKI is characterized by acute deterioration of kidney function and disruption of electrolyte and fluid homeostasis, which occur within a few hours or days. AKI is associated with increased long-term risks of poor clinical outcomes, including chronic kidney disease (CKD), cardiovascular disease, and mortality [23,24]. Currently, the commonly used biomarker of renal function is serum creatinine (SCr) [25]. Creatinine is a waste product in the blood that is generated from muscle activity. Creatinine is normally removed from the blood by the kidneys. However, the creatinine levels increase with the deterioration of kidney function. AKI is characterized by elevated SCr levels within 7 days or by a sustained reduction in urine output over 6 h [25]. However, Scr is not a sensitive marker for early AKI event, and, therefore, the development of more sensitive, accurate, and cost-effective biomarker assays for clinical AKI assessment other than Scr analysis is crucial [26]. In addition to SCr, several urine biomarkers have emerged as more sensitive indicators for early detection of AKI, such as urine levels of kidney injury molecule-1 (KIM-1), neutrophil gelatinase-associated lipocalin (NGAL), activin A, and insulin-like growth factor-binding protein 7 (IGFBP-7) [27,28,29,30]. Urine Biomarker [(Tissue inhibitor of metalloproteinases 2) TIMP-2] × [IGFBP-7] level has also been proposed as a promising indicator for clinical AKI risk assessment [31].

One of the major challenges for AKI treatment is the heterogeneity of the disease. The pathophysiology of AKI varies according to the different conditions associated with its development [32]. AKI could be induced by drugs, renal toxins, sepsis, glomerulonephritis, or acute ischemic reperfusion [32]. Traditionally, maintenance of adequate renal vascular perfusion through volume and hemodynamic management remains the major medical treatment for AKI, as well as avoiding nephrotoxins and drugs associated with kidney injury [33]. Renal replacement therapy can be implemented while awaiting clinical signs of renal function recovery [32]. However, an effective therapy for preventing acute kidney injury, apart from supportive management, remains a largely unmet medical need.

Following acute kidney injury, an initial phase involving cell damage or cell death occurs in accordance with the specific nature of the insults. This phase lasts from minutes to hours and involves epithelial, endothelial, and other renal parenchymal cells in certain specific or even all zones of the kidneys [34]. At this stage, the damaged tissue/cells release a broad range of danger signals for initiating an acute inflammatory response. Following the primary insult, a phase of tissue inflammation is initiated and mediated predominantly by tissue-resident and infiltrating immune cells including neutrophils and macrophages [35]. The recruited immune cells promote the clearance of dead cell debris and trigger anti-inflammation responses by secreting anti-inflammatory factors to promote inflammation resolution and tissue repair [35]. Renal peritubular fibroblasts are activated and promote extracellular matrix production for tissue repair [36]. However, maladaptive repair or excessive interstitial myofibroblast activation may result in excessive extracellular matrix (ECM) deposition and worsen renal function recovery [37]. The tissue reparative process could last for long periods (days to weeks) after acute tissue damage depending on the type of injury insults, reparative capability, efficiency of dead cell clearance by phagocytes, and resolution of inflammatory responses [38]. In a clinical study, more than 80% of the patients who experienced an AKI episode recovered their renal function within the first week, while approximately 17% recovered after more than a week [39]. The mechanism underlying successful and maladaptive tissue repair, as well as excessive fibrosis remains to be completely elucidated.

To better understand the underlying molecular mechanism and the contribution of immune cells in the progression of AKI, several animal models of AKI have been established. The most common mouse models of AKI that have been generated include ischemia-reperfusion injury (IRI), chemical-induced injury (doxorubicin or cisplatin), and UUO [40,41,42]. These animal models have helped improve our understanding of tissue responses to acute kidney insults and the involvement of different types of immune cells or cytokines in the inflammatory and reparative phases [35]. However, the experimental design for assessing the outcome may affect the interpretation of the results. Therefore, there is a need to comprehensively analyze the dynamic changes in tissue reparative responses, immune cell profiles, fibrosis markers, and soluble serum markers for evaluating kidney injury and to gain an in-depth understanding of the detailed mechanism underlying the regulation of inflammatory process at different phases of disease onset.

Several studies have demonstrated that both innate and adaptive immune responses contribute to tissue inflammation post-kidney injury [43]. However, the mechanism underlying the regulation of tissue inflammation and inflammation resolution is unclear. Cytokines are reported to be involved in the progression of renal fibrosis and tissue damage [44]. IL-33 is reported to promote type 2 immune responses and allergic diseases in mucosal tissues, such as lung, intestine, and skin [45]. However, IL-33 also promotes anti-inflammatory responses to suppress the inflammation via alternatively activated macrophages (AAMs), myeloid-derived suppressive cells (MDSCs), and regulatory T-cells (Tregs) [46,47]. Recent studies have also demonstrated that IL-33-mediated immune responses are involved in renal protection using mouse AKI models [48,49,50,51]. The growth factors, such as amphiregulin (AREG) derived from IL-33-responsive group 2 innate lymphoid cells (ILC2s) or Tregs, are reported to promote epithelium regeneration after tissue injury [52]. In addition to the renal protective functions in AKI models, recent studies have reported the detrimental effects of IL-33, which involve promoting renal fibrosis [53,54,55]. This review summarizes the current findings on IL-33-mediated immune responses in kidney injury, repair, and fibrosis.

## 2. Roles of IL-33-Mediated ILC2 Activation in the Kidney

Several studies have reported the pathological roles of IL-33 in renal IRI [53], cisplatin-induced AKI [20], and ovalbumin-induced nephrotoxicity models [56]. Additionally, some recent studies have demonstrated that IL-33-mediated immune responses contribute to renal protection in the mouse AKI models [48,49,50,51]. The IL-33/ST2 signaling pathway may function as a double-edged sword and is involved in both the pathological and tissue reparative processes by affecting various cell types at different phases of disease progression [18]. The recent findings on the characterization of kidney IL-33-responsive immune cells, especially ILC2s, in kidney injury are discussed below.

### 2.1. Characterization of IL-33-Responsive Kidney ILC2s

The tissue ILC subsets are largely characterized by flow cytometric analysis of the cell surface markers, nuclear transcription factors, or intracellular cytokine expression [57]. Riedel et al. analyzed the distribution of ILC subsets in the human and mouse kidneys [48]. The flow cytometry analysis revealed that the total ILCs (Lin-CD127+CD161+) comprise CRTH2+ ILC2s, NKp44+, and NKp44-CRTH2-CD117+ ILC3s, while a small population of CRTH2-CD117-NKp44- ILC1s reside in the normal human kidney [48]. The study also identified that the normal mouse kidney has Lin-GATA3+ ILC2s and small populations of RORγt+ ILC3s and Tbet+ILC1s. In the normal mouse kidney, ILC2s constitute 80% of total ILC subsets [48]. Upon IL-33 treatment (0.4 µg per day for 4 consecutive days, intraperitoneal (i.p.)), the kidney ILC2s expand and sustain for up to 13 weeks. The renal ILC2s express the IL-33 receptor, ST2, and CD25 in the healthy human and mouse kidneys [48]. The tissue localization was further validated by immunofluorescent staining using suitable antibodies for different surface or nuclear antigens [48]. The immunofluorescent staining analysis revealed that the kidney ILC2s are localized in the glomerular and tubulointerstitial compartment around the peritubular capillaries [48]. Cao et al. also analyzed the distribution of kidney ILC2s using flow cytometry, which revealed that IRI alone did not alter the number of kidney ILC2 and that the treatment with exogenous IL-33 enhances the ILC2 populations [49].

Our previous study had analyzed the ST2+ immune cell profile in the unilateral urinary obstruction (UUO) mouse model [58]. The study reported that kidney ILC2s (CD45+Lin-Thy1.2+GATA3+) constitute the major population (approximately 70%) among the total ILC subsets in the normal and injured kidneys [58]. In addition to kidney ILC2s, other ST2-expressing immune populations were also identified. Among the ST2+ cells, the CD45- non-leukocytes, neutrophils, macrophages, T/ILCs, and monocytes comprised 34.8%, 22.3%, 23.1%, 16.2%, and 3.6%, respectively [58]. Compared with the nonobstructed kidneys, the UUO kidneys exhibited a 30-fold increase in the number and a 4.6-fold increase in the percentage of ST2+ neutrophils [58]. These results indicate that there is increased infiltration and expansion of ST2+ innate immune cells in the kidney following obstructive injury.

Cameron et al. comparatively analyzed the phenotypes of kidney and lung ILC2s using flow cytometry analyses [59]. The analyses revealed that the kidney ILC2s exhibited lower CD25 expression, consistent expression of inducible T cell costimulator (ICOS) and killer cell lectin like receptor G1 (KLRG1), and upregulated IL-5 expression when compared with the lung ILC2s [59]. The study used the Rosa-tdtomato lineage tracker to label the IL-5-expressing cells and reported that the kidney ILC2s are localized around the vasculature under homeostatic conditions [59]. Additionally, the renal ILC2s were reported to be the major IL-5-producing cells in the kidney [59]. Intriguingly, reduction, deficiency, or depletion of ILC2s does not alter the severity of kidney injury in mice following IRI. This suggested that other type 2 immune responses may compensate for the absence of ILC2s [59]. In humans, the lack of ILCs might be compensated by other T-cell and B-cell preserved functions [60]. The ILC2s present in the human kidney [48] may be a potential therapeutic target for treating kidney diseases. However, the role of activated kidney-resident ILC2s in the renal protective response in humans is unclear.

An increasing body of evidence revealed the presence of ILC2s residing in the healthy kidneys of humans and mice (Table 1). The regulation of ILC2 expansion by exogenous IL-33 may elicit the renal protective responses in mouse AKI models (Table 2). IL-33 has been shown to promote the egress of bone marrow ILC2s [61]. Since exogenous IL-33 activated systemic responses, it is not restricted in activation of kidney ILC2s. However, it is not clear whether IL-33-promoted ILC2 activation in other organs contributes to the renal protective functions via increasing the systemic serum factors. Also, it is not clear whether the IL-33-activated bone marrow or spleen ILC2s migrate and accumulate in the injured kidneys. The precise pathophysiological functions of the kidney-resident ILC2s in homeostasis and disease conditions remain largely unknown.

### 2.2. Recent Findings on Exogenous IL-33-Mediated ILC2s Activation in Kidney Injury Models

In addition to the pro-inflammatory function, IL-33 enhances pulmonary tissue repair responses via ILC2s [63]. Recent studies have also revealed that IL-33-mediated activation of ILC2 cells, Tregs, and AAMs may have protective roles in renal injury. In the adriamycin-induced glomerulosclerosis model, treatment with exogenous IL-33 results in sustained expansion of ST2^+^ ILC2, which ameliorates the renal injury through eosinophils [48]. Huang et al. demonstrated that IL-25-responsive ILC2 and type 2 multipotent progenitor (MPP^type2^) cells promoted macrophage polarization toward the M2 phenotype in the kidney and prevented acute renal IRI [64]. Cao et al. demonstrated that treatment with exogenous IL-33 protects the mice against IRI (pre-treatment or post-treatment). Treatment with exogenous IL-33 decreased the number of GR-1+ myeloid cells post-IRI and induced Th2 cytokines, ILC2s, Tregs, and AAMs in vivo. Interestingly, Cao et al. reported that Treg did not contribute to the IL-33-mediated renoprotection in IRI mice. GW2580-mediated AAM depletion or anti-CD90-mediated ILC depletion abolished the IL-33-mediated renoprotection in IRI mice [49]. Adoptive transfer of ILC2 protects the mice from IRI, which is mediated by AREG [49]. These results suggest that exogenous IL-33-mediated ILC2 activation reduces tissue inflammation and promotes tissue repair following IRI.

Recently, Sharma et al. combined IL-2 and IL-33 for fine-tuning the immunomodulatory effects of IL-33 on Tregs and ILC2s [51]. The hybrid IL-2–IL-33 (IL233) fusion protein exhibited promising therapeutic efficacy in various mouse models of kidney injuries, including IRI [51], doxorubicin-induced nephrotoxicity [50], lupus nephritis [65], and diabetic nephropathy [66]. These studies indicated that the IL233 fusion protein could be a novel immunomodulatory cytokine that regulates multiple nephropathic factors with potential renoprotective applications. Whether IL233 could be applied to other disease models could be interesting and require further investigation. These recent studies suggest that exogenous IL-33-mediated ILC2 activation has a role in protection against AKI in mice.

Although the IL-33 protein levels are upregulated in the kidneys after AKI, the kidney-resident ILC2s are not accumulated or expanded unless exogenous IL-33 was systemically administered [48,49,50,51]. This potentially implied that the endogenous IL-33 may not be sufficient to elicit the expansion of kidney ILC2s. However, the local elevation in the IL-33 protein levels may be sufficient to mediate the immunomodulatory function, which promotes neutrophil infiltration and amplifies the tissue innate inflammation during the acute inflammatory phase of AKI. The exogenous IL-33-mediated expansion of ILC2s and Tregs may skew the tissue immune microenvironment toward the tissue reparative responses that favor inflammation resolution and regeneration. We summarized the key findings of recent studies on the effect of endogenous and exogenous IL-33 in AKI models (Table 2).

In contrast to the short-term administration of IL-33, the prolonged systemic IL-33/ST2 signaling activation might be detrimental after the recovery of damaged tissue. IL-33-mediated immunosuppression is reported to be associated with sepsis-induced immunosuppression [67]. The role of exogenous IL-33-induced immunosuppression in inducing global immunosuppression at the later stage of tissue recovery in the IRI models remains unclear and must be elucidated for optimizing the timing and duration of IL-33-based therapy.

## 3. Roles of Endogenous IL-33 Versus Exogenous IL-33 in Kidney Injury, Repair, and Fibrosis

Genetically modified mice lacking endogenous IL-33 or its receptor (ST2) have been used for the loss-of-function study to examine the role of IL-33/ST2 signaling in the pathogenesis of AKI. Ferhat et al. demonstrated that endogenous IL-33 contributes to the recruitment of myeloid cells upon acute IRI using *IL33*-deficient mice [62]. IL-33 is constitutively expressed on blood vessels, especially peritubular and periglomerular endothelial cells [20,62,68]. Ferhat et al. also demonstrated that endogenous IL-33 is not required for early recruitment of myeloid cells post-IRI (1–6 h) but is required for the amplification of neutrophil-mediated inflammatory response via invariant natural killer T-cell (iNKT) [62]. Based on these results, Ferhat et al. concluded that endogenous IL-33 functions as a tissue alarmin and contributes to kidney IRI via IL-33-mediated iNKT activation and enhances neutrophil recruitment following AKI [62].

Akcay et al. revealed that treatment with high-dose exogenous IL-33 (1 µg, twice a day on days 1, 2, and 3 after cisplatin administration, i.p. injection) exacerbates cisplatin-induced AKI via CD4 T-cells/CXCL1 axis in the mouse model [20]. Administration of soluble ST2 (sST2; 100 µg/day, on days 1, 2, and 3 of cisplatin administration) decreased the levels of SCr, acute tubular necrosis, and tubular apoptosis [20]. The dose of IL-33 used in this study was higher than that used in other recent studies [48,49,50,51]. It is not clear if the detrimental effect of exogenous IL-33 observed in this study resulted from the excessive immune response elicited by IL-33. A recent study by Edelstein and colleagues reported that *IL33* deficiency does not protect against cisplatin-induced AKI in mice with cancer [69]. In contrast to their earlier study that used acute high-dose cisplatin, this study used four weeks low-dose cisplatin to induce AKI. The effect of *IL33* deficiency at the early time point (within one day post-cisplatin administration) was not analyzed. Hence, it is currently unknown whether the endogenous IL-33 contributes only to early inflammatory phase and is dispensable for the progression of sustained renal injury in this model.

In addition to the renal protective functions of IL-33, several recent studies also revealed the profibrotic role of IL-33 in the IRI and UUO models [19,54,55]. Liu et al. demonstrated that BRG1 (Brahma-related gene-1)-mediated IL-33 expression promotes profibrogenic responses in the endothelial and renal tubular cells and that the knockdown of BRG-1 in the endothelial cells mitigated the ischemia/reperfusion-induced renal injury and fibrosis in the mouse model [54]. Liang et al. demonstrated that treatment with exogenous IL-33 (0.5 µg/day for 2 weeks; i.p. injection) promoted IRI-induced renal fibrosis, whereas IL-33 neutralization by sST2 (100 µg/day for 2 weeks; i.p. injection) decreased the degree of kidney injury, extracellular matrix depositions, myeloid fibroblast accumulation, myofibroblast formation, and infiltration of T-cells and macrophages in the kidneys at day 14 post-IRI [53]. In this study, the contralateral kidney was removed at day 5 after unilateral IRI surgery to evaluate the renal function post-IRI [53].

UUO model has been extensively used to study the progression of renal fibrosis following kidney obstruction [42,70]. In the UUO model, the obstructed kidney is characterized by renal tubular dilation, loss of proximal tubular mass, infiltration of immune cells, and fibrosis. Upon obstructive kidney injury, the insults of mechanical stretch and increased intra-renal pressure may lead to progressive renal tubulointerstitial fibrosis if the obstruction is not resolved [42]. Our previous study reported that the deficiency of *IL33* or *IL1RL1* partially reversed the UUO-induced renal fibrosis [19]. Similarly, Li et al. demonstrated that UUO kidney exhibited upregulated expression levels of *IL33* and *IL1RL1*. Exogenous IL-33 increased the macrophage infiltration and renal fibrosis with a concomitant increase in the AAMs [55]. Li et al. reported that exogenous IL-33 promotes kidney fibrosis via the effector function of AAMs in the UUO model [55]. Our previous study investigated the ST2+ immune cell profile in the kidney, which revealed that the myeloid cells are the major targets of IL-33 in the UUO-induced obstructed kidney [58]. However, the deletion of *Il33* reduced the protein levels of type 2 cytokines in the urine isolated from the UUO kidney [58]. This suggested that the endogenous IL-33 contributes to the production of the type 2 cytokines in the UUO model. The differential roles of IL-33 in tissue fibrotic responses can be attributed to the different pathological conditions, tissue responses upon injury insults, and the analysis endpoints between IRI and UUO models. Additionally, the UUO model potentially exhibits both acute and chronic insults following irreversible kidney obstruction and is attributed to differential pathological outcomes between the IRI and UUO models.

## 4. Potential Applications and Caveats of Therapeutic Targeting IL-33/ST2 Signaling

In the last few decades, the loss-of-function approaches, such as gene knockout or specific immune cell depletion models, have been extensively used to investigate the biological function of IL-33/ST2 axis-mediated immunomodulatory response and its therapeutic potential. It is important to note that the interpretations of results from current studies using *IL33* knockout (endogenous) or administration of IL-33 (exogenous) could vary depending on the use of different models. Additionally, IL-33 activity might be regulated by the endogenous antagonist, sST2. However, it is unclear whether the local tissue sST2 concentration is involved in the regulation of IL-33-mediated immune responses in vivo. The beneficial effects of *IL33* deficiency and application of exogenous IL-33 or sST2 have been demonstrated by different groups. However, it is important to note that the experimental findings using exogenous IL-33 administration do not equally explain the endogenous function of IL-33 as IL-33 is endogenously expressed in various organs. The local concentration of mature IL-33 should be considered when interpreting the results from studies using exogenous IL-33 treatment.

IL-33 knockout mouse was used to analyze the loss-of-function of endogenous IL-33. It remains unclear whether the dual roles of the nuclear IL-33 or extracellular IL-33 via ST2-independent or ST2-dependent pathways contribute to differential outcomes under disease conditions, such as IRI. In the IL-33-deficient mice, both the nuclear and extracellular functions of IL-33 were abolished. Although the exact function of nuclear IL-33 is unclear, the nuclear IL-33 was reported to interact with nuclear factor (NF)-κB and inhibit NF-κB-stimulated gene transcription [71]. The nuclear function and stability of IL-33 were regulated by the enzyme ubiquitin-specific protease 17 (USP17) through the deubiquitination of IL-33 at both the K48 and K63 sites [72]. The biological activity of IL-33 is rapidly inactivated in the extracellular environment by forming two disulfide bonds, which results in a conformational change and disruption of the ST2 binding site [73]. The release of IL-33 from the nucleus to the extracellular matrix was mediated by an ATP-dependent nuclear pore complex [74]. The chromatin binding of IL-33 was reported to be associated with the ST2 signaling in neutrophil extracellular traps (NET)-induced signaling [75]. However, the detailed molecular mechanisms involved in the regulation of IL-33 translocation in living cells are poorly understood. The nuclear expression of IL-33 was dispensable for its biological function in the human umbilical vein endothelial cells (HUVECs) [76]. In addition to the endothelial cells, other types of cells express nuclear IL-33 in the kidney, such as interstitial fibroblasts [19] and hyperplasic KRT-5+ urothelial cells [58]. The effect of *IL33* deficiency on gene expression in these cells under healthy and disease conditions remains unknown.

Several groups have generated *IL1RL1*-deficient mice for the genetic deletion of the IL-33 receptor, which is encoded by the *IL1RL1* gene. Sehnine et al. used the *IL1RL1* knockout mice to demonstrate that streptozotocin (STZ)-induced hyperglycemic mice exhibited an increased urinary albumin excretion [77]. At 24 h post-IRI, the ST2 knockout mice exhibited markedly reduced plasma creatinine, blood urea nitrogen (BUN), and tubular injury. However, this protective effect was mitigated in the ST2-induced diabetes mellitus (DM) mice [77]. This suggested that the high glucose environment may impair the IL-33/ST2-mediated renal protective function. Several groups have generated *ST2* knockout mice by knocking out the *IL1RL1* gene. The *IL1RL1* knockout in mice resulted in the deletion of both membrane (ST2L) and soluble (sST2) forms of ST2. However, the production of sST2 and the formation of sST2 concentration gradient, which is observed in the myocardium, in the kidney are unknown [78]. Additionally, the role of IL-33/ST2 signaling in non-immune cells is not well understood. Osmotic stress regulates the expression of *IL1RL1* in the collecting duct cells. However, the type of transcript isoform that is altered is not known [79]. The comparative analysis of IL-33 and sST2 tissue levels, as well as the distribution of ST2-expressing cells (immune and non-immune cells), will aid in understanding the mechanistic control of IL-33/ST2 signaling within the tissue microenvironment. Moreover, the *IL1RL1* knockout lines currently used lack both the ST2L and sST2 isoforms, which prevents the interpretation of the physiological regulation of IL-33/ST2 signaling by sST2. Future studies must focus on specifically targeting the ST2L or sST2 isoforms, which will improve our understanding of the IL-33/ST2 axis-mediated immune responses.

## 5. Conclusions

**Figure 1:**
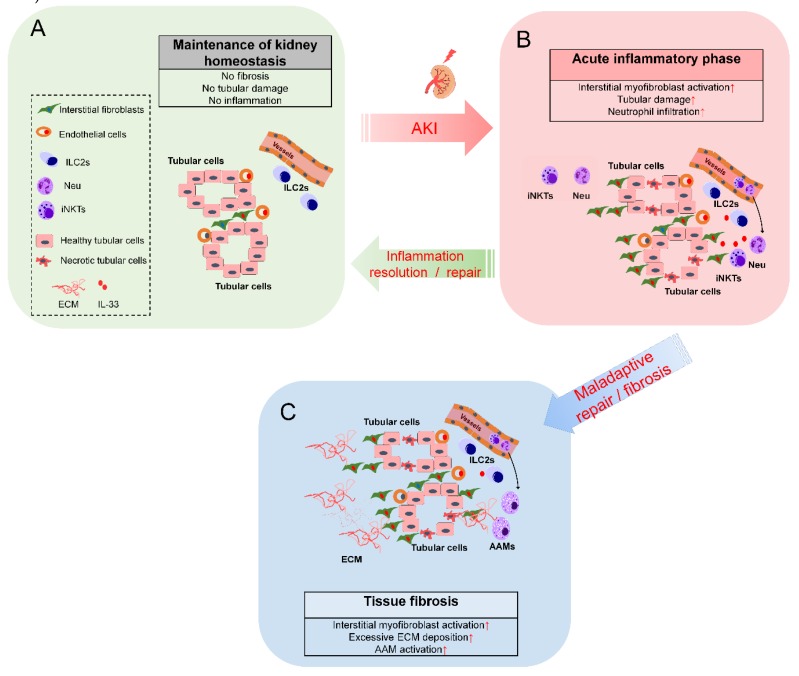
IL-33-mediated immunomodulatory networks in acute kidney injury. (**A**) IL-33 is expressed in the interstitial fibroblasts and endothelial cells in healthy kidney. (**B**) During the acute inflammatory phase of following kidney injury (0–24 h), IL-33 mediates the recruitment of immune cells, such as neutrophils and macrophages, and the amplification of inflammatory responses via iNKT cells. (**C**) During the recovery phase, IL-33 can mediate the activation of anti-inflammatory responses via ILC2s, alternatively activated macrophages (AAMs), and regulatory T-cells (Tregs). However, maladaptive repair or excessive interstitial myofibroblast activation may result in excessive extracellular matrix (ECM) deposition. Long-term treatment with high-dose exogenous IL-33 may promote tissue fibrosis if the analysis is performed at later stages of disease onset following kidney injury.

**Figure 2:**
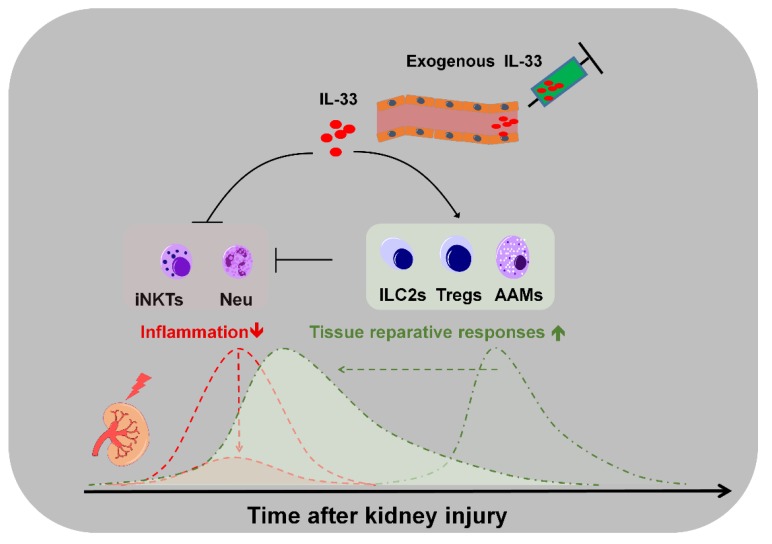
IL-33 treatment may promote tissue-reparative responses and inhibit inflammation in AKI. Treatment with exogenous IL-33 at different time points before or after kidney injury can mediate differential immune responses by targeting different immune cells. IL-33 treatment may skew the kidney ILC2s toward renal protective response. Additionally, IL-33 treatment following ischemia-reperfusion injury (IRI) could promote inflammation resolution and kidney repair potentially through ILC2- and AAM-derived factors. The renal protective function of exogenous IL-33 is potentially mediated by AAMs in the absence of endogenous ILC2s.

Recent evidence indicates that IL-33/ST2 signaling contributes to the pathogenesis of multiple diseases that are associated with kidney injury. Short-term exogenous IL-33 treatment at the early stage of disease onset could be beneficial, whereas long-term (high-dose) treatment may exacerbate the progression of renal fibrosis. The tissue-resident ILC2s and Tregs could be the first-line immune microenvironment modifiers in response to exogenous IL-33 treatment. Further studies are needed to elucidate the IL-33-mediated (or ILC2- and Tregs-mediated) immune microenvironment changes in the kidney for treating AKI. This review summarized the potential functions of endogenous IL-33- and exogenous IL-33-mediated tissue responses in kidney tissues following acute injury (Figure 1 and Figure 2).

Collectively, IL-33 exhibits diverse immune regulatory functions during the various phases of different diseases. Endogenous IL-33, which is upregulated at early stages after tissue injury, functions as an acute-phase alarmin to amplify the innate inflammatory responses, whereas exogenous IL-33 (short-term, low-dose) can function as anti-inflammatory via activation of ILC2s, Tregs, and MDSCs. The immunomodulatory roles of IL-33 in tissue inflammation and repair in different renal injury models are not completely understood and require comprehensive investigation.

In clinical settings, sepsis in critically ill patients is one of the major causes of AKI. IL-33 has been shown to promote bacteria clearance in a mouse model of sepsis [80]. However, it is not clear whether IL-33 treatment contributes to improved kidney function in the septic animals. Additionally, the balance between neutrophil- and eosinophil-mediated immune responses have attributed to the outcomes of ILC2-mediated protective effect against *Staphylococcus aureus* bacteremia-induced mortality [80]. Further investigation to determine blood neutrophils versus eosinophils ratio in patients with sepsis-associated AKI will help to decipher the contribution of the IL-33/ILC2 axis in the patient population.

The tissue reparative responses and the process of tissue fibrosis are double-edged swords. The fine-tuning of tissue reparative process could result in less fibrotic scar formation and promote optimal tissue repair and clinical outcomes. Currently, an effective therapy for preventing acute kidney injury, other than supportive management, remains a largely unmet medical need. IL-33 may function as a crucial immunomodulator to mediate the balance between tissue repair and fibrosis responses and could be a potential therapeutic target for AKI. Although IL-33 has been shown to play a protective role in several AKI animal models, there are several limitations to the use of these AKI models in the study of inflammation and therapeutic targets. These limitations include the difference in species, immune responses, and susceptibility to AKI insults between humans and animal models [34]. For clinical applications, further studies are needed to understand the IL-33-mediated immune responses in humans. The optimization of the timing, dosages, and tissue specificity may be crucial for the development of IL-33-based therapeutics for treating various kidney diseases, such as AKI.

## Figures and Tables

**Table 1:** Characterization of kidney group 2 innate lymphoid cells (ILC2s).

Species	ILC Subsets (Markers)	Characteristics	Tissue Localization	Reference
Human	Total ILCs (Lin-CD127+CD161+); ILC2s (Lin-CD127+CD161+CRTH2+); ILC3s (NKp44+, and NKp44-CRTH2-CD117+); ILC1s (CRTH2-CD117-NKp44-)	* ILC3 constitute ~55% of total ILCs; * ILC2 constitute ~30% of total ILCs; * ILC1 constitute ~10% of total ILCs		Riedel et al. [48]
Mouse	ILC2s CD45+Lin-CD127+CD90.2+ST2+	*Kidney ILC2s constitutively express IL-5	IL-5+ILC2s localize the major vasculature in healthy kidney	Cameron et al. [59]
Mouse	ILC2s (CD45+Lin-CD127+GATA3+	* Mouse ILC2s constitute ~80% of total ILC2s	ILC2s localized in glomerular and tubulointerstitial compartments	Riedel et al. [48]
Mouse	ILC2s (CD45+Lin-CD127+GATA3+ST2+)	* IRI alone did not altered ILC2s		Cao et al. [49]
		* Exogenous IL-33 expands ILC2s		
Mouse	ILC2s (CD45+Lin-ST2+)	* Adoptive transfer of ex vivo expanded spleen-derived ILC2s by IL-233 hybrid protects the against IRI		Stremska et al. [51]
Mouse	ILC2s (CD45+Lin-ST2+CD90+)	* IL233 expands spleen and kidney ILC2s		Sabapathy et al. [50]
Mouse	ILC2s (CD45+Lin-CD90.2+ GATA3+)	* Mouse ILC2s constitute ~70% of total ILCs;		Chen et al. [58]
		* ILC2s were expended 2 fold following UUO		

ILC, innate lymphoid cells; CRTH2, chemoattractant receptor-homologous molecule expressed on TH2 cells; Lin, lineage, GATA3, GATA binding protein 3; IRI, ischemia-reperfusion injury.

**Table 2:** Effects of endogenous and exogenous IL-33 in AKI models.

**Animals**	**Model**	**Key Findings**	**Reference**
**Endogenous IL-33**			
*IL33* ^Gt/Gt^	IRI	Capillary CD31+ endothelial cells are the source of IL-33	Ferhat et al. [62]
		IL-33 knockout is protective to IRI	
		Reduced myeloid cell infiltration in IL-33 knockout mice	
		Impaired iNKT recruitment and function in the IL-33 knockout mice	
		Early IL-33 release (1–6 h post injury) is not necessary for myeloid recruitment after IRI	
		IL-33-mediated iNKT activation contributes to neutrophil recruitment during the amplification phase of kidney injury	
**Exogenous IL-33 treatment**			
mouse IL-33 (0.3 μg/mice for 5 days); C57BL/6	IRI	Exogenous IL-33 protects mice against IRI (pre-treatment or post-treatment)	Cao et al. [49]
		Exogenous IL-33 decreased GR-1+ myeloid cells post-IRI	
		Exogenous IL-33 induced Th2 cytokines, ILC2s, Tregs, and AAMs in vivo	
		IL-33 boosts kidney-resident ILC2 proliferation in vivo	
		Treg did not contribute to IL-33-mediated renoprotection in IRI mice	
		GW2580-mediated AAM depletion abolished IL-33-mediated renoprotection in IRI mice	
		Anti-CD90-mediated ILC depletion abolished IL-33-mediated renoprotection in IRI mice	
		Adoptive transfer of ILC2 protects mice from IRI	
		AREG mediates renoprotective function of ILC2s	
		IL-33 and ILC2s are protective post-IRI	
mouse IL233 (66pmol /mice for 5 days); C57BL/6J and Balb/cJ	IRI Doxorubicin Cisplatin	Exogenous IL-2 combined with IL-33 (IL233) is protective post-IRI	Stremska et al. [51]; Sabapathy et al. [50]
		IL233 promotes the expansion of ILC2s and Tregs, which is required for the renal protective functions	
		IL233 is renal protective in IRI-, cisplatin-, and doxorubicin-induced nephrotoxicity	
mouse IL-33	IRI	Exogenous IL-33 promotes IRI-induced fibrosis	Liang et al. [53]
		sST2 attenuates IRI-induced renal injury and fibrosis	
mouse IL-33 (0.4μg/mice for 4 days); C57BL/6 and Balb/c	Adriamycin (Doxorubicin)	ST2+ILC2s are the major ILC2 population in human and mouse kidneys	Riedel et al. [48]
		Exogenous IL-33 induces sustained ILC2 expansion in the kidneys	
		Exogenous IL-33 ameliorates Adrimycin-induced kidney injury	
		Eosinophils are required for IL-33-mediated tissue protection	

AKI, acute kidney injury; IRI, ischemia-reperfusion injury; ILC2, group 2 innate lymphoid cells, AAM, alternative activated macrophages; iNKT, invariant nature killer T-cells; sST2, soluble ST2.

## Abbreviations

AAMs	alternatively activated macrophages
AKI	acute kidney injury
AREG	amphiregulin
BUN	blood urea nitrogen
CKD	chronic kidney disease
DM	diabetes mellitus
ERK	extracellular signal-regulated kinase
ECM	extracellular matrix
IGFBP-7	insulin-like growth factor-binding protein 7
ILC2	group 2 innate lymphoid cells
IL-33	Interleukin-33
IL-1RacP	IL-1 receptor accessory protein
HUVECs	human umbilical vein endothelial cells
iNKT	invariant natural killer T-cell
IL1RL1	IL-1 receptor like 1
IRAK-1	IL-1 receptor-associated kinase-1
IRI	ischemia-reperfusion injury
JNK	c-Jun N-terminal kinases
KIM-1	kidney injury molecule-1
MDSCs	myeloid-derived suppressive cells
MyD88	myeloid differentiation primary response 88
NF-κB	nuclear factor kappa B
NGAL	neutrophil gelatinase-associated lipocalin
PIN1	peptidyl-prolyl cis-trans isomerase NIMA-interacting 1
SIGIRR	single Ig IL-1-related receptor
SCr	serum creatinine
TLR	toll-like receptors
TIR	toll-interleukin receptor
TIMP-2	tissue inhibitor of metalloproteinases 2
Tregs	regulatory T-cells
UUO	unilateral urinary obstruction
